# Treatment of Chronic Mammary Abscess by the Breast Pump and Syringe

**Published:** 1845-08

**Authors:** 


*Treatment of Chronic Mammary Abscess by the Breast Pump and Syringe.*—Dr. Alexander Wood proposes to treat that form of mammary abscess which gives rise to deep-seated sinuses, and sometimes continues for many months with little variation in its appearance, in the following manner: "As soon as the indistinct fluctuation, or rather the boggy feeling, by which the formation of matter in these abscesses can be detected, is distinctly ascertained, let a small bistoury or abscess lancet (the common lancet will sometimes not penetrate deep enough) be carried down until the matter begins to escape; after all that can be squeezed out by pressure is removed, let a breast-pump be applied over the orifice, and the rest of the matter drawn out. The sinus is then to be injected with some astringent solution by means of a small syringe." "A pledget of lint dipped in the lotion is then to be applied outside, and covered with oiled silk; over this a compress may be placed, and firm pressure maintained on it by means of adhesive plaster. In some cases the walls of the abscess will unite at once, and all that remains to be done is to trust to time for the removal of the surrounding induration, or to attempt to discuss it by frictions, &c. &c. Dr. Wood adduces three cases of chronic abscess in which he adopted this mode of treatment with complete and speedy success. We can imagine that this plan would be found serviceable in various forms of abscess. It is always observed that, where the pus lies deep, and is but imperfectly evacuated, the disease proves intractable; the great indication for cure appears to be, that all irritating fluid should be removed from the depths of the cavities, and that the internal vascular surfaces should be brought closely into apposition with each other.—*Northern Jour. Med.—Bul. Med. Sci. for July.*

